# Misoprostol Administration Before Hysteroscopy Procedures – A Retrospective Analysis

**DOI:** 10.1055/s-0042-1755462

**Published:** 2022-08-29

**Authors:** Quênya Antunes Silveira Inácio, Júlia Kefalás Troncon, Fernando Passador Valério, Helmer Herren, Antônio Alberto Nogueira, Omero Benedicto Poli Neto, Júlio César Rosa e Silva

**Affiliations:** 1 Departamento de Ginecologia e Obstetrícia, Faculdade de Medicina de Ribeirão Preto, Universidade de São Paulo, Ribeirão Preto, SP, Brazil

**Keywords:** hysteroscopy, misoprostol, complications, side effects, histeroscopia, misoprostol, complicações, efeitos colaterais

## Abstract

**Objective** To evaluate the use of misoprostol prior to hysteroscopy procedures regarding technical ease, the presence of side effects, and the occurrence of complications.

**Methods** This is a retrospective, observational, analytical, case-control study, with the review of medical records of 266 patients followed-up at the Gynecological Videoendoscopy Sector of the Hospital das Clínicas da Faculdade de Medicina de Ribeirão Preto of the Universidade de São Paulo (HCFMRP – USP, in the Portuguese acronym) from 2014 to 2019, comparing 133 patients who used the drug before the procedure with 133 patients who did not.

**Results** The occurrence of postmenopausal uterine bleeding was the main indication for hysteroscopy and revealed a statistical difference between groups ( *p* < 0.001), being present in 93.23% of the patients in the study group and in 69.7% of the patients in the control group **.** Only 2 patients (1.5%) in the study group reported adverse effects. Although no statistical differences were observed regarding the occurrence of complications during the procedure ( *p* = 0.0662), a higher total number of complications was noted in the group that used misoprostol ( *n* = 7; 5.26%) compared with the group that did not use the drug ( *n* = 1; 0.75%), a fact that is clinically relevant. When evaluating the ease of the technique (measured by the complete performance of all steps of the hysteroscopy procedure), it was verified that although there was no difference between groups ( *p* = 0.0586), the control group had more than twice as many incompletely performed procedures ( *n* = 17) when compared with the group that used misoprostol previously ( *n* = 8), which is also clinically relevant.

**Conclusion** The use of misoprostol prior to hysteroscopy in our service indicated that the drug can facilitate the performance of the procedure, but not without side effects and presenting higher complication rates.

## **Resumo**

**Objetivo** Avaliação do misoprostol prévio à histeroscopia quanto à facilidade técnica, efeitos colaterais e a ocorrência de complicações durante o procedimento.

**Métodos** Estudo analítico observacional retrospectivo tipo caso controle com revisão de prontuários de 266 pacientes do Setor de Videoendoscopia Ginecológica do Hospital das Clínicas da Faculdade de Medicina de Ribeirão Preto - Universidade de São Paulo (HCFMRP – USP), de 2014 a 2019, sendo comparadas 133 pacientes que utilizaram o medicamento prévio ao procedimento com 133 pacientes que não o utilizaram.

**Resultados** Sangramento uterino após a menopausa foi a principal indicação de histeroscopia, apresentando diferença estatística ( *p* < 0,001), estando presente em 93,23% das pacientes do grupo de estudo e em 69,17% das pacientes do grupo controle. Apenas 2 pacientes (1,5%) do grupo de estudo relataram efeitos adversos. Não foram observadas diferenças quanto à presença de complicações durante o procedimento ( *p* =  0,0662), mas observamos um número total de complicações maior no grupo de estudo ( *n* = 7; 5,26%) do que no grupo controle ( *n* = 1; 0,75%), o que é clinicamente relevante. Não houve diferença entre os grupos quanto à facilidade técnica ( *p* = 0,0586), mas o grupo controle apresentou mais do que o dobro de procedimentos não completamente realizados ( *n* = 17) quando comparado com o grupo de estudo ( *n* = 8), o que é clinicamente relevante.

**Conclusão** O uso de misoprostol prévio à histeroscopia no nosso serviço demonstrou que ele pode facilitar a realização do procedimento, mas não é isento de efeitos colaterais e apresenta maiores taxas de complicações.

## Introduction

The hysteroscopic procedure emerged ∼ 200 years ago, providing the direct visualization of diffuse or focal uterine abnormalities, the anatomical configuration of the cervical canal and the uterine cavity, path permeability, access for biopsies, and direct removal of lesions. 1 2 3 It is considered a minimally invasive procedure, and its use is quite common both in the diagnosis and in the treatment of several conditions, such as abnormal uterine bleeding, the evaluation of infertile patients, surgeries including myomectomy and polypectomy, the diagnosis of endometrial and endocervix hyperplasia and carcinoma, among others, often being performed in clinics or as outpatient follow-up procedures. 1 2 3 4 5 This method has an important advantage over other diagnostic techniques: the anatomopathological confirmation of lesions visually identified through guided biopsy. 4

In order to carry out the procedure, it is often necessary to dilate the cervix, especially in surgical hysteroscopies in which the equipment for performing the procedure is larger than the endocervical canal. 6 The most frequent causes of unsatisfactory exams are cervical stenosis, pain or patient intolerance, bleeding that hinders hysteroscopic view, and technical difficulties. 7 In an attempt to reduce these technical problems and the number of unsatisfactory exams, several methods of cervical dilation have been developed over the years, such as the use of hydrophilic laminators, bladder catheter balloons, and Hegar dilators. These techniques, however, cause great discomfort to patients and increase the risk of complications during the dilation process. 3 4 5 8 The complication rate varies between 0.3 and 5% according to the definition used, the most common being pain, vagal reaction, uterine perforation, false passage formation, and cervical lacerations. Serious complications, such as organ perforation and pelvic infection, are seldom reported. 7 9

Therefore, there was a need to develop new methods of cervical dilation, which should include cervix preparation for a limited time, acceptable for the patient, with ease of administration, quick action, and providing adequate cervical ripening to facilitate the procedure. Misoprostol is one of the most studied agents in this context. 10 It is a synthetic analog of prostaglandin E1 that has been used for cervical preparation prior to performing hysteroscopy because it promotes effective cervical ripening, as well as being an inexpensive, easy to store and administer, and widely available method. 4 5 The most common adverse effects of misoprostol occur mainly before the procedure and include cramping, abdominal and/or pelvic pain, nausea, changes in intestinal transit, vaginal bleeding of varying intensity, and fever and/or chills. However, these effects are generally described as tolerable and rarely motivate the cancellation or alteration of the procedure. 11 12

According to the scientific literature, misoprostol is effective in cervical ripening in the preoperative period of hysteroscopy, reducing the time needed for cervical dilation and increasing the mean cervical diameter. Nevertheless, the optimal dose, the route of administration, and the ideal time of administration prior to hysteroscopy, in addition to whether the drug reduces the rates of pre- and postmenopausal complications, remain unclear, a fact that justifies the importance of carrying out the present study in daily gynecological practice, whose objectives were to assess the ease of the operative hysteroscopic technique with the use of misoprostol, evaluated by the complete performance of the steps of the procedure, to assess the presence of side effects with the use of the drug, and to analyze the occurrence of hysteroscopic complications with its use. 5 8

## Methods

The present analytical, observational, case-control study was conducted by reviewing the medical records of patients followed up at the Gynecological Videoendoscopy Sector of the HCFMRP – USP in the period from 2014 to 2019. Using a list provided by the Medical Support Service (SAME, in the Portuguese acronym), a total of 508 patients with prescriptions for the drug misoprostol for intrahospital use at the HCFMRP– USP were identified in the analyzed period. By cross-referencing the data of patients followed-up at the Gynecological Videoendoscopy Sector of the same hospital and who had used misoprostol prior to hysteroscopy, 207 patients were identified. The medical records of these 207 patients were reviewed, evaluating: age, parity, type of delivery, time since menopause, associated diseases (systemic arterial hypertension [SAH], type 2 diabetes mellitus [DM2], obesity, and other comorbidities), use of continuous medication, symptoms, postmenopausal bleeding, presence of endometrial thickening, side effects of the drug, procedure complications, and complete performance of the procedure. Among the total patients, 74 lacked data in their medical records or had their hysteroscopy procedures suspended for various reasons unrelated to the use of misoprostol or to the procedure itself and were therefore excluded. The 133 patients included in the study were compared with another 133 age-matched patients also followed-up at the Gynecological Videoendoscopy Section of the HCFMRP – USP who underwent hysteroscopy procedures but who did not use misoprostol previously (control group), regarding the ease of the technique, considered easy when all steps of the hysteroscopy procedure were carried out completely, the presence of side effects, and the occurrence of complications. Patients who used misoprostol before the hysteroscopy did so by vaginally introducing 2 tablets of 200 micrograms (µg) each the night before the procedure, totaling a single dose of 400 µg.

After filling out the study database, which evaluated age, parity, type of delivery, time since menopause, associated diseases (SAH, DM2, obesity, and other comorbidities), the use of continuous medication, symptoms, postmenopausal bleeding, presence of endometrial thickening, drug side effects, procedure complications, and its complete performance, a statistical analysis was carried out using SPSS Statistics for Windows, Version 17.0 (SPSS Inc., Chicago, IL, USA). The distribution of the variables was assessed using the Kolmogorov-Smirnov test. Normally-distributed data were analyzed with the *t* -test, while abnormally distributed data were evaluated using the Kruskal-Wallis test. Mean values were presented with statistical significance (SS), which was accepted for *p* < 0.05. Numerical data were presented as mean ± standard deviation (SD) or median and range, depending on their distribution. The chi-squared test was used for variables expressed as percentages, considering a significance level of *p* < 0.05.

Since this was a retrospective study involving medical record analysis, the Research Ethics Committee of the HCRP – USP was asked to waive the application of informed consent through a Letter of Exemption from the collection of the consent form, given most of the patients were no longer being followed-up at the hospital. The present study, as well as the waiver of written informed consent, were approved by the Research Ethics Committee (REC) of the HCRP – USP on February 17, 2020, under CAAE Protocol No. 28983920.0.0000.5440. All ethical precepts were followed as recommended by the Helsinki Convention.

## Results

Regarding the clinical characteristics of the studied patients, no significant difference was observed between those who used misoprostol or not prior to the hysteroscopy procedure in relation to age ( *p* = 0.9005), the number of pregnancies ( *p* = 0.4586), the number of vaginal deliveries ( *p* = 0.5531), the time since menopause ( *p* = 0.9193), history of previous cesarean delivery ( *p* = 0.8723), or regarding the number of prior abortions ( *p* = 0.8528) ( Tables 1 and 2 ). When analyzing the comorbidities presented by the patients at the time of hysteroscopy, we observed a significant difference in relation to SAH ( *p* = 0.0041), but not regarding DM2 ( *p* = 0.0622), obesity ( *p* = 0.5082), or other comorbidities ( *p* = 0.3510). The fact that the patients used continuous medications for these comorbidities also did not differ significantly between the 2 groups ( *p* = 0.3023) ( Table 2 ).

**Table 1:** Clinical characteristics of the studied population

	With misoprostol	Without misoprostol	*p* -value
	Mean ± SD	Mean ± SD	
Age (years old)	60.08 ± 8.5	59.95 ± 8.15	0.9005
Pregnancy	3.44 ± 1.96	3.24 ± 2.47	0.4586
Vaginal delivery	2.13 ± 2.17	1.96 ± 2.37	0.5531
Time since menopause	10.98 ± 7.48	11.08 ± 8.22	0.9193

Abbreviation: SD, standard deviation.

**Table 2:** Clinical characteristics of the studied population

	With misoprostol		Without misoprostol		*p* -value
	*n* (133)	%	*n* (133)	%	
Cesarean delivery					0.8723
0	61	45.86	69	51.88	
1	33	24.81	27	20.30	
2	23	17.29	23	17.29	
3	15	11.28	13	9.77	
4	1	0.75	1	0.75	
Miscarriage					0.8528
0	99	74.44	99	74.44	
1	24	18.05	23	17.29	
2	8	6.02	6	4.51	
3	1	0.75	2	1.50	
4	1	0.75	2	1.50	
5	0	0.00	1	0.75	
SAH					0.0041
Yes	111	83.46	91	68.42	
No	22	16.54	42	31.58	
DM2					0.0622
Yes	63	47.37	48	36.09	
No	70	52.63	85	63.91	
Obesity					0.5082
Yes	44	33.08	39	29.32	
No	89	66.92	94	70.68	
Other comorbidities					0.3510
Yes	89	66.92	96	72.18	
No	44	33.08	37	27.82	
Continuous use of medications					0.3023
Yes	127	95.49	123	92.48	
No	6	4.51	10	7.52	
Menopause					1.0000
Yes	123	92.48	123	92.48	
No	10	7.52	10	7.52	
Symptoms					<0.001
Yes	124	93.23	96	72.18	
No	9	6.77	37	27.82	
Postmenopausal bleeding					<0.001
Yes	124	93.23	92	69.17	
No	9	6.77	41	30.83	
Endometrial thickening					0.6419
Yes	124	93.23	122	91.73	
No	9	6.77	11	8.27	

Abbreviations: DM2, diabetes mellitus type 2; *n* , number of samples; SAH, systemic arterial hypertension.

Both groups had the same number of patients before and after menopause, with no difference in hormonal status in relation to the use of misoprostol ( *p* = 1.000). The presence of symptoms reported by the patients for the indication of the hysteroscopic procedure showed a difference between the 2 groups ( *p* < 0.001); 93.23% of the patients in the group that used misoprostol had at least 1 symptom versus 72.18% of the patients in the control group. Among the most reported symptoms was postmenopausal uterine bleeding, which also showed a significant difference between the 2 groups ( *p* < 0.001); 93.23% of the patients in the group that used misoprostol had this symptom versus 69.17% of the patients in the control group. Meanwhile, asymptomatic endometrial thickening showed no statistically significant difference between the 2 groups ( *p* = 0.6419). Regarding the adverse effects observed in the patients who used the drug prior to hysteroscopy, only 2 patients (1.5%) reported symptoms: both presented tremors, and 1 presented with symptoms of anxiety. Although no significant difference was observed in relation to the occurrence of complications during the procedure ( *p* = 0.0662), a higher total number of complications was observed in the group that used misoprostol ( *n* = 7; 5.26%) compared with the group that did not ( *n* = 1; 0.75%), which is clinically relevant. In the control group, the only reported complication was false passage formation. Meanwhile, in the group that used misoprostol, the most frequent complication was the absence of uterine cavity distension, which was observed in three of the patients. The other observed complications included cervical laceration ( *n* = 1), uterine perforation ( *n* = 2), and increased fluid absorption ( *n* = 1), the latter 2 of which compelled the termination of the procedure. In the group of patients who used misoprostol, false passage formation was not reported ( Table 3 ).

**Table 3:** Complications reported after the hysteroscopy procedure

	With misoprostol		Without misoprostol		*p* -value
	*n* (133)	%	*n* (133)	%	
Complications					0.0662
Yes	7	5.26	1	0.75	
No	126	94.74	132	99.25	
Uterine Cervical Laceration					0.9999
Yes	1	0.75	0	0	
No	132	99.25	133	100	
Absence of uterine cavity distension					0.2619
Yes	3	2.26	0	0	
No	130	97.74	133	100	
Uterine perforation					0.5079
Yes	2	1.50	0	0	
No	131	98.50	133	100	
Increased fluid absorption					0.9999
Yes	1	0.75	0	0	
No	132	99.25	133	100	
False passage					0.9999
Yes	0	0	1	0.75	
No	133	100	132	99.25	

Abbreviation: *n* , number of samples.

Finally, upon evaluating the technical ease (performing all steps of the procedure), we noted that although there was no difference between groups ( *p* = 0.0586), the control group had more than twice as many incompletely performed procedures ( *n* = 17) when compared with the group that used misoprostol previously ( *n* = 8), which is clinically relevant.

## Discussion

The present study compared 133 women who used misoprostol prior to hysteroscopy with 133 who did not use the medication. This drug is a prostaglandin E1 analog oxytocin that causes changes in the physicochemical structure of cervical collagen. After its administration, misoprostol undergoes de-esterification in the liver into misoprostolic acid. This active metabolite exerts direct action on prostaglandin receptors, leading to the softening and ripening of the cervix, favoring its dilation, in addition to promoting an increase in intracellular calcium, which is responsible for the contraction of uterine muscles. 13 All of these mechanisms enable progressive cervical effacement and dilation. 4 5

The systematic review of misoprostol suggests variations in the plasma concentration of this drug depending on the route of administration. Orally, the drug is rapidly and completely absorbed in the gastrointestinal tract; however, it is also quickly and extensively metabolized into its acidic form in the first hepatic pass (de-esterification). A single 400-µg dose of oral misoprostol reaches its peak concentration in 30 minutes and declines in ∼ 120 minutes, remaining at a low level. After vaginal administration, on the other hand, there is a gradual increase in the plasma concentration of misoprostol, reaching a maximum level after 70 to 80 minutes, followed by a slow decline, with the drug level still detectable after 6 hours. It has also been reported that the mean concentration peak via the sublingual route is higher than that achieved via the oral and vaginal routes, which is due to the rapid absorption by the sublingual mucosa, avoiding first-pass metabolism in the liver. When administered rectally, the absorption curve of the drug is similar to that seen when using the vaginal route. 14

Corroborating another study carried out with 77 women between January 2005 and March 2006, we did not observe a significant difference in age and the number or type of previous births between the study group and the control group. 15 Although no significant difference was found in the present study regarding pre- and postmenopausal patients, some studies suggest that the use of misoprostol is more effective in dilating the cervix of premenopausal patients, mainly due to the hormonal difference between these women. 4 Regarding the comorbidities presented by the patients at the time of the procedure, only SAH showed significance.

In our study, postmenopausal uterine bleeding was the most prevalent symptom, observed in 93.23% of the patients in the study group and in 69.17% of those in the control group. A case review coordinated by Gimpelson et al. 16 revealed a high incidence of abnormal bleeding, which was observed in 76% of the cases as the chief complaint of patients to undergo hysteroscopy. In addition, a descriptive, cross-sectional study with 26 women showed that the primary complaint of 65.3% of the patients was uterine bleeding. 17

The occurrence of adverse effects to the use of misoprostol was reported by only 2 patients (1.5%), who presented tremors and anxiety. A meta-analysis evaluating 14 studies showed that significantly more adverse effects were reported when misoprostol was administered compared with procedures without previous use of the drug (odds ratio [OR] = 3.56; 95% confidence interval [CI]: 1.60–7.93). 18 Two other studies described the incidence of adverse effects among women randomized to 200 or 400 μg of misoprostol, but their data were conflicting: the first did not demonstrate a dose-related increase in adverse effects (nausea and abdominal pain) in women randomized to 200 or 400 μg ( *p* = 1.0 and *p* = 0.055, respectively); however, the second study showed a significant increase in the number of adverse events related only to 400 μg ( *p* = 0.015), such as fever, abdominal pain, diarrhea, nausea, vomiting, and vaginal bleeding. 18 Regarding the administration time, these studies showed that there was no significant difference in the incidence of abdominal cramps ( *p* = 0.64), nausea ( *p* = 0.79), diarrhea ( *p* > 0.99), genital tract bleeding ( *p* = 0.62), and fever ( *p* > 0.99) among women who received misoprostol 12 hours or 3 hours before hysteroscopy. 18 One study with 160 women comparing oral, sublingual, and vaginal administration of the drug concluded that the 3 groups were comparable, and all the adverse effects were similar in all groups and were tolerable. This result is in line with a recent meta-analysis that analyzed 7 randomized, controlled studies involving 568 individuals, evaluating the use of misoprostol in surgical hysteroscopy. Compared with the placebo group, there was an increase in side effects (cramps, vaginal bleeding, nausea, and diarrhea) in the misoprostol group (relative risk [95%CI]: 4.28 [1.43­–12.85]). 19 20

The incidence of complications in our study was low compared with the mean described in the literature. 9 A total of 7 complications occurred in patients who had previously used misoprostol (5.26%) and in 1 patient who did not use the medication (0.75%). The primary complication reported among patients who used misoprostol was the absence of uterine cavity distension, which occurred in 3 patients. This was also found by Batukan et al. 15 in a randomized, double-blind, placebo-controlled study carried out with the objective of evaluating the efficacy of 400 µg of misoprostol 10 to 12 hours before surgical hysteroscopy in premenopausal women, via the vaginal route. One of the reported disadvantages of vaginal administration was excessive cervical dilation, resulting in difficulty distending the uterine cavity due to fluid leakage through the cervical canal. 8 Another study, prospectively conducted between January 2005 and March 2006 at the Department of Obstetrics and Gynecology of the Faculty of Medicine of Erciyes University, with 77 women, showed that vaginal administration of misoprostol (400 μg) prior to operative hysteroscopy in premenopausal women was superior to the same dose of orally administered misoprostol. The complication rates during cervical dilation, as well as drug side effects, were comparable between the two regimens. Fluid leakage caused by excessive cervical dilation and effacement appeared to be the most important potential disadvantage of vaginal misoprostol. Since the intrauterine pressure did not reach the ideal desired level in these cases, the uterine distension was suboptimal and, therefore, the procedure was more difficult. 15

In the present study, the other complications observed in patients who used misoprostol were cervical laceration ( *n* = 1; 0.75%), uterine perforation ( *n* = 2; 1.5%), and increased absorption of distension media ( *n* = 1; 0.75%). The patient who did not use misoprostol presented false passage formation as a complication. In a study carried out at Bringham and Women's Hospital, located in Boston, MA, USA, Propst et al. 21 verified a total number of 925 surgical hysteroscopies between 1995 and 1996, with the occurrence of complications in 2.7% of the patients. Among these complications was cervical laceration, which was present in all cases. In another study carried out by Jansen et al., 22 in which 13,600 hysteroscopies in 82 hospitals in the United States were evaluated in 1997, as well as in the study by Agostini et al., 23 performed in Marseille, France, in which 2,116 surgical hysteroscopies between 1990 and 1999 were analyzed, the most frequent complication among the surgical procedures was uterine perforation, with 0.76% and 1.61% of cases, respectively. 22 23 The study by Propst et al. 21 also reported complications related to uterine distension media in 1.4% of the surgical hysteroscopy cases. In a randomized, controlled, double-blind study conducted by Oppengard et al., 24 in which each participant received 1,000 µg of misoprostol or placebo, which was self-inserted vaginally at least 12 hours before operative hysteroscopy, there were a total of 9 (11%) complications reported.

Regarding technical ease, which was evaluated by the complete performance of all steps of the procedure, we observed that although there was no statistically significant difference, the number of patients who did not use misoprostol and did not undergo the complete procedure was more than double in relation to the patients who used the medication (8 patients who used the drug versus 17 patients who did not), which is of substantial clinical relevance. Fernandez et al. 25 reported in a larger series that the administration of 400 µg of oral misoprostol 12 or 24 hours before surgery or 200 µg of vaginal misoprostol 9 to 10 hours before surgery, respectively, demonstrated greater ease of cervical dilation. However, to date, no placebo-controlled trials have shown a significant decrease in the rate of serious complications such as cervical laceration or perforation. 25

The prospective study conducted between January 2005 and March 2006 at the Department of Obstetrics and Gynecology of the Faculty of Medicine of Erciyes University showed that vaginally administered misoprostol (400 µg) prior to operative hysteroscopy in premenopausal women was superior to the same dose of orally administered misoprostol in terms of shorter cervical dilation and surgery duration, as well as the need for cervical dilation for No. 9 Hegar. 15 Other previous studies comparing patients who used misoprostol or placebo previously to prepare the cervix, as evidenced by Uckuyu et al. 26 in Ankara, Turkey, also show a relevant rate of failure in dilation with Hegar dilators, especially in patients who had had previous cesarean sections. In the aforementioned study, the reported failure rate of cervical dilation using Hegar dilators was 25%. 26

Our study had some limitations, such as the absence of a patient-reported pain assessment, given most of the analyzed patients underwent the procedure in the operating room under anesthesia; lack of evaluation of different doses and routes of administration of misoprostol, since all of the patients used the drug at a dose of 400 µg via the vaginal route; the absence of evaluation of the time of misoprostol administration, seeing that, in all patients, the drug was introduced the night before the procedure. Costa et al. 27 conducted a randomized study with 120 postmenopausal women who received 200 µg of vaginal misoprostol or placebo 8 hours before outpatient hysteroscopy. There was a significant reduction in the pain scale during the procedure, a fact that would facilitate the performance of outpatient surgical procedures. In 2018, Fouda et al. 28 evaluated the effect of timing of vaginal misoprostol administration (3 hours versus 12 hours) before diagnostic hysteroscopy in nulliparous women, who are at increased risk for cervical canal stenosis. The group of women given the drug just 3 hours before hysteroscopy reported more pain during the procedure than those given the medication 12 hours earlier. However, the pain intensity 30 minutes after the procedure, its mean duration, and the occurrence of side effects to misoprostol were similar between the 2 groups. The passing of the hysteroscope through the cervical canal was also assessed by the examiners and was found to be easier in the 12-hour group.

## Conclusion

The use of misoprostol prior to hysteroscopy in our service showed that the drug can facilitate the performance of the procedure; however, this drug is not free from side-effects and higher complication rates. Also, misoprostol is a well-tolerated drug. We agree with most authors that there is a need for further studies to better identify the ideal dose, route of administration, and time to indicate misoprostol before the procedure.

## References

[1] Martins F N Martins N N Histeroscopia diagnóstica [Internet] Coimbra Federação das Sociedades Portuguesas de Obstetrícia e Ginecologia 2010[cited 2021 Dec 12]. p. 359-71. Available from:http://www.fspog.com/fotos/editor2/cap_42.pdf

[2] Ganer Herman H Kerner R Gluck O Feit H Keidar R Bar J Different routes of misoprostol for same-day cervical priming prior to operative hysteroscopy: a randomized blind trial J Minim Invasive Gynecol 2017 24 03 455 460 10.1016/j.jmig.2016.12.024 28069480

[3] Valente E P Uso de misoprostol vaginal para redução da dor em histeroscopia diagnóstica na menacme: ensaio clínico randomizado, triplamente mascarado, controlado com placebo [dissertação] Recife Instituto Materno Infantil Professor Fernando Figueira 2017

[4] Bastu E Celik C Nehir A Dogan M Yuksel B Ergun B Cervical priming before diagnostic operative hysteroscopy in infertile women: a randomized, double-blind, controlled comparison of 2 vaginal misoprostol doses Int Surg 2013 98 02 140 144 10.9738/INTSURG-D-12-00024.1 23701149 PMC3723170

[5] Zhuo Z Yu H Jiang X A systematic review and meta-analysis of randomized controlled trials on the effectiveness of cervical ripening with misoprostol administration before hysteroscopy Int J Gynaecol Obstet 2016 132 03 272 277 10.1016/j.ijgo.2015.07.039 26797202

[6] Fradique A Histeroscopia cirúrgica [Internet] Coimbra Federação das Sociedades Portuguesas de Obstetrícia e Ginecologia 2010[cited 2021 Dec 12]. p. 511-33. Available from:http://www.fspog.com/fotos/editor2/cap_49.pdf

[7] Clark T J Voit D Gupta J K Hyde C Song F Khan K S Accuracy of hysteroscopy in the diagnosis of endometrial cancer and hyperplasia: a systematic quantitative review JAMA 2002 288 13 1610 1621 10.1001/jama.288.13.1610 12350192

[8] Allen R O'Brien B M Uses of misoprostol in obstetrics and gynecology Rev Obstet Gynecol 2009 2 03 159 168 19826573 PMC2760893

[9] Costa A A Estudo randomizado, duplamente mascarado, placebo controlado do uso do misoprostol versus placebo para histeroscopia diagnóstica em mulheres na pós-menopausa [tese] Campinas Universidade Estadual de Campinas 2006

[10] Moreira A R O uso de misoprostol em histeroscopia: revisão do seu papel em contexto diagnóstico e cirúrgico [monografia] Porto Universidade do Porto 2018

[11] de Albuquerque L G Hardy E Bahamondes L Histerossonografia: avaliação da cavidade uterina com sangramento anormal Rev Assoc Med Bras (1992) 2006 52 04 247 250 10.1590/S0104-42302006000400025 16967143

[12] Angioni S Loddo A Milano F Piras B Minerba L Melis G B Detection of benign intracavitary lesions in postmenopausal women with abnormal uterine bleeding: a prospective comparative study on outpatient hysteroscopy and blind biopsy J Minim Invasive Gynecol 2008 15 01 87 91 10.1016/j.jmig.2007.10.014 18262151

[13] Koch D M Rattmann Y D Uso do misoprostol no tratamento da hemorragia pós-parto: uma abordagem farmacoepidemiológica Einstein (Sao Paulo) 2020 18 eAO5029 10.31744/einstein_journal/2020AO5029 31721897 PMC6896658

[14] Souza A S Amorim M M Costa A A Noronha Neto C Farmacocinética e farmacodinâmica do misoprostol em obstetrícia Femina 2019 37 12 679 684

[15] Batukan C Ozgun M T Ozcelik B Aygen E Sahin Y Turkyilmaz C Cervical ripening before operative hysteroscopy in premenopausal women: a randomized, double-blind, placebo-controlled comparison of vaginal and oral misoprostol Fertil Steril 2008 89 04 966 973 10.1016/j.fertnstert.2007.03.099 17681307

[16] Gimpelson R J Rappold H O A comparative study between panoramic hysteroscopy with directed biopsies and dilatation and curettage. A review of 276 cases Am J Obstet Gynecol 1988 158(3 Pt 1):489 492 10.1016/0002-9378(88)90011-7 3348309

[17] Bezerra Júnior A L Costa A A Fernandes G C Santos J V Pereira T M Resultados intra e pós-operatórios e a frequência de complicações em pacientes pré e pós-menopausadas submetidas à histeroscopia cirúrgica no IMIP [tcc] Recife Instituto Materno Infantil de Pernambuco 2019

[18] De Silva P M Wilson L Carnegy A Smith P P Clark T J Cervical dilatation and preparation prior to outpatient hysteroscopy: a systematic review and meta-analysis BJOG 2021 128 07 1112 1123 10.1111/1471-0528.16604 33219606

[19] Selk A Kroft J Misoprostol in operative hysteroscopy: a systematic review and meta-analysis Obstet Gynecol 2011 118 04 941 949 10.1097/AOG.0b013e31822f3c7b 21934459

[20] Song T Kim M K Kim M L Jung Y W Yoon B S Seong S J Effectiveness of different routes of misoprostol administration before operative hysteroscopy: a randomized, controlled trial Fertil Steril 2014 102 02 519 524 10.1016/j.fertnstert.2014.04.040 24856464

[21] Propst A M Liberman R F Harlow B L Ginsburg E S Complications of hysteroscopic surgery: predicting patients at risk Obstet Gynecol 2000 96 04 517 520 10.1016/s0029-7844(00)00958-3 11004351

[22] Jansen F W Vredevoogd C B van Ulzen K Hermans J Trimbos J B Trimbos-Kemper T C Complications of hysteroscopy: a prospective, multicenter study Obstet Gynecol 2000 96 02 266 270 10.1016/s0029-7844(00)00865-6 10908775

[23] Agostini A Bretelle F Cravello L Ronda I Roger V Blanc B [Complications of operative hysteroscopy] Presse Med 2003 32 18 826 829 12870384

[24] Oppengard K S Nesheim B I Istre O Qvigstad E Comparison of self-administered vaginal misoprostol versus placebo for cervical ripening prior to operative hysteroscopy using a sequential trial design BJOG 2008 115 05 663,e 1–9 10.1111/j.1471-0528.2007.01628.x 18201279 PMC2345467

[25] Fernandez H Alby J D Tournoux C Chauveaud-Lambling A DeTayrac R Frydman R Vaginal misoprostol for cervical ripening before operative hysteroscopy in pre-menopausal women: a double-blind, placebo-controlled trial with three dose regimens Hum Reprod 2004 19 07 1618 1621 10.1093/humrep/deh302 15155607

[26] Uckuyu A Ozcimen E E Sevinc F C Zeyneloglu H B Efficacy of vaginal misoprostol before hysteroscopy for cervical priming in patients who have undergone cesarean section and no vaginal deliveries J Minim Invasive Gynecol 2008 15 04 472 475 10.1016/j.jmig.2008.03.001 18588848

[27] Costa HdeL Costa L O [Hysteroscopy in menopause: analysis of the techniques and accuracy of the method] Rev Bras Ginecol Obstet 2008 30 10 524 530 10.1590/s0100-72032008001000008 19082390

[28] Fouda U M Elsetohy K A Elshaer H S Hammad B EM Shaban M Youssef M A Misoprostol prior to diagnostic office hysteroscopy in the subgroup of patients with no risk factors for cervical stenosis: a randomized double-blind placebo-controlled trial Gynecol Obstet Invest 2018 83 05 455 460 10.1159/000480234 28982101

